# Cardiorespiratory fitness and cardiometabolic health are associated with distinct cognitive domains in cognitively healthy older adults

**DOI:** 10.1038/s41598-025-26105-x

**Published:** 2025-11-28

**Authors:** Patrycja Kałamała, Nicholas Ware, Monica Fabiani, Patricia Michie, Montana Hunter, Alexandra Wade, Felicity Simpson, Maddison L. Mellow, Kathy Low, Hannah A. D. Keage, Gabriele Gratton, Ashleigh E. Smith, Frini Karayanidis

**Affiliations:** 1 Centre for Cognitive Science, Jagiellonian University, Gołębia 24, Kraków, 31-007 Poland; 2 Beckman Institute for Advanced Science and Technology, University of Illinois Urbana-Champaign, Urbana-Champaign, USA; 3 Functional Neuroimaging Laboratory, School of Psychological Sciences, College of Engineering, Science and the Environment, University of Newcastle, University Dr, Callaghan, Newcastle, NSW 2308 Australia; 4 Hunter Medical Research Institute, Newcastle, NSW Australia; 5 Department of Psychology, University of Illinois Urbana-Champaign, Urbana-Champaign, USA; 6 School of Psychology and Vision Sciences, University of Leicester, Leicester, UK; 7 Alliance for Research in Exercise, Nutrition and Activity (ARENA), Allied Health and Human Performance, University of South Australia, Adelaide, South Australia Australia; 8 Behaviour-Brain-Body Research Centre, Justice and Society, University of South Australia, Adelaide, South Australia Australia

**Keywords:** Aging, Cognition, Cardiometabolic health, Cardiorespiratory fitness, Executive functions, Crystallized ability, Cardiology, Health care, Physiology

## Abstract

Cardiometabolic risk and low cardiorespiratory fitness have been linked to age-related cognitive decline, but their relative contributions to different cognitive domains remain unclear. This study examined cross-sectional associations between cardiometabolic health, cardiorespiratory fitness, and cognition in cognitively healthy older adults (60–70 years). Structural equation modelling (SEM) was used to estimate latent variables for cardiorespiratory fitness (derived from physical activity, resting heart rate and BMI), cardiometabolic health (derived from blood pressure, glucose, lipids and BMI), and four cognitive domains: processing speed, executive function, verbal memory, and crystallized ability. Better cardiorespiratory fitness was significantly associated with higher executive function and processing speed, but not with verbal memory or crystallized ability. In contrast, better cardiometabolic health was significantly associated with higher crystallized ability and verbal memory, but not with executive function or processing speed. These modest but reliable relationships remained significant when both health factors were included in the same model, suggesting a double dissociation between their cognitive correlates. This study provides the first evidence that cardiorespiratory fitness and cardiometabolic health are related to distinct aspects of cognition in later life, highlighting potential targets for lifestyle-based interventions to support cognitive health in older age.

## Introduction

Healthy aging is associated with structural and functional changes in the brain as well as subtle declines in a range of cognitive abilities^1,2^. Given the brain’s high energy demands, it is not surprising that these brain and cognitive changes are associated with changes in cardiovascular health that become more prevalent with increasing age.

Cardiovascular health is characterized by a range of physiological factors used to estimate cardiometabolic health and cardiorespiratory fitness. These are closely interrelated aspects of physiological functioning that influence brain and cognitive aging. Both cardiometabolic health and cardiorespiratory fitness typically decline with increasing age, and the rate of decline is influenced by genetic predisposition, lifestyle, and disease processes^3–5^.

Cardiometabolic health reflects vascular and metabolic integrity and is commonly indexed using composite risk scores. Several established formulas are used in research and clinical settings to quantify cardiometabolic risk, including the Framingham Risk Score and the Metabolic Syndrome criteria (e.g., metabolic syndrome severity score, MetSSS)^6–9^. These indices typically incorporate variables such as systolic and diastolic blood pressure, fasting glucose, high-density lipoprotein cholesterol (HDL), triglycerides, and body mass index (BMI), capturing the key physiological pathways that contribute to cardiometabolic burden. Such formulas provide practical tools for summarizing complex health profiles and are widely used in studies of aging and cognition. Low cardiometabolic health is associated with increased risk of cardiovascular and associated diseases^10^ and greater risk of vascular dementia^11^.

Poor cardiometabolic health is also associated with poorer performance in cognitive domains that are sensitive to aging^12–14^. These include executive functions (i.e., processes that regulate and coordinate cognitive resources to support goal-directed behavior), processing speed (i.e., the efficiency with which basic cognitive operations are executed), and, to a lesser degree, verbal memory (i.e., the ability to learn, retain, and retrieve verbal information). In contrast, crystallized abilities (i.e., accumulated knowledge and vocabulary) are relatively spared^15–18^. Individual risk factors contributing to cardiometabolic health are associated with poorer cognitive performance (e.g., hypertension)^19,20^.

Cardiorespiratory fitness reflects the body’s capacity to perform sustained physical work and depends on the integrated function of cardiovascular, respiratory, and musculoskeletal systems^19,20^. Although maximal exercise testing (e.g., VO₂Max) remains the gold standard, cardiorespiratory fitness can be reliably estimated using non-exercise algorithms, such as the Jurca score, which combine readily obtainable variables including age, sex, BMI, resting heart rate, and self-reported physical activity. These scalable models are well suited to large epidemiological and non-clinical samples and demonstrate high predictive validity (*R²* = 0.60–0.85)^21,22^. On average, cardiorespiratory fitness declines by approximately 8–10% per decade in healthy adults, with reductions of up to 50% by age 80 relative to mid-life^23,24^.

Lower cardiorespiratory fitness is also consistently linked to poorer performance in fluid cognitive domains, with weaker or no associations observed for crystallized abilities^25,26^. Higher cardiorespiratory fitness is also associated with better cognitive outcomes longitudinally^25,27^. Notably, enhancing cardiorespiratory fitness has been shown to mitigate some age-related declines in brain structure and cognitive function^28–33^. In contrast, evidence supporting cognitive improvements through cardiometabolic health interventions is currently limited.

A recent paper by España-Irla et al.^34^ is the first to compare how cardiometabolic health and cardiorespiratory fitness relate to different cognitive domains. Using data from the Barcelona Brain Health Initiative (BBHI; *n* = 501, aged 40–65 years), they found that higher cardiometabolic risk was correlated with poorer performance across multiple cognitive domains. In contrast, cardiorespiratory fitness was more specifically associated with visuospatial and problem-solving abilities, but only in the older age range (55–65 years). In addition, whole brain cortical thickness mediated the relationship between cardiometabolic health and cognition. The relationship between cardiorespiratory fitness and cognition was mediated more specifically by cortical thickness in frontal regions, again in the older subgroup. However, the study did not directly examine whether these two dimensions of cardiovascular health have unique domain-specific associations with cognitive functioning. As cardiorespiratory fitness and cardiometabolic health are interrelated^35,36^, their shared variance may have masked distinct relationships with cognition. Controlling for their shared variance can clarify these unique contributions and reveal whether different physiological pathways - such as those related to vascular integrity versus aerobic efficiency - selectively support distinct cognitive domains. This information can guide targeted strategies to maintain cognitive health in older age.

In the present study, we directly address this gap by examining the common and unique associations of cardiorespiratory fitness and cardiometabolic health with four key cognitive domains in a large cohort of community-dwelling adults aged 60–70 years^1,37^. The cognitive domains included crystallized abilities, which remain largely preserved in healthy older adults^37^, as well as verbal memory, executive functioning, and processing speed - domains that are particularly sensitive to aging^1^.

Cardiometabolic health was operationalized as a latent variable using biological measures (i.e., systolic and diastolic blood pressure, fasted glucose, HDL and triglycerides) and anthropometric indicators (i.e., BMI). This aligns with established clinical risk assessments like the Framingham Risk Score, which includes these key variables^38–40^. Cardiorespiratory fitness was operationalized as a distinct latent variable based on validated fitness estimators^21,41^.

**Fig. 1:**
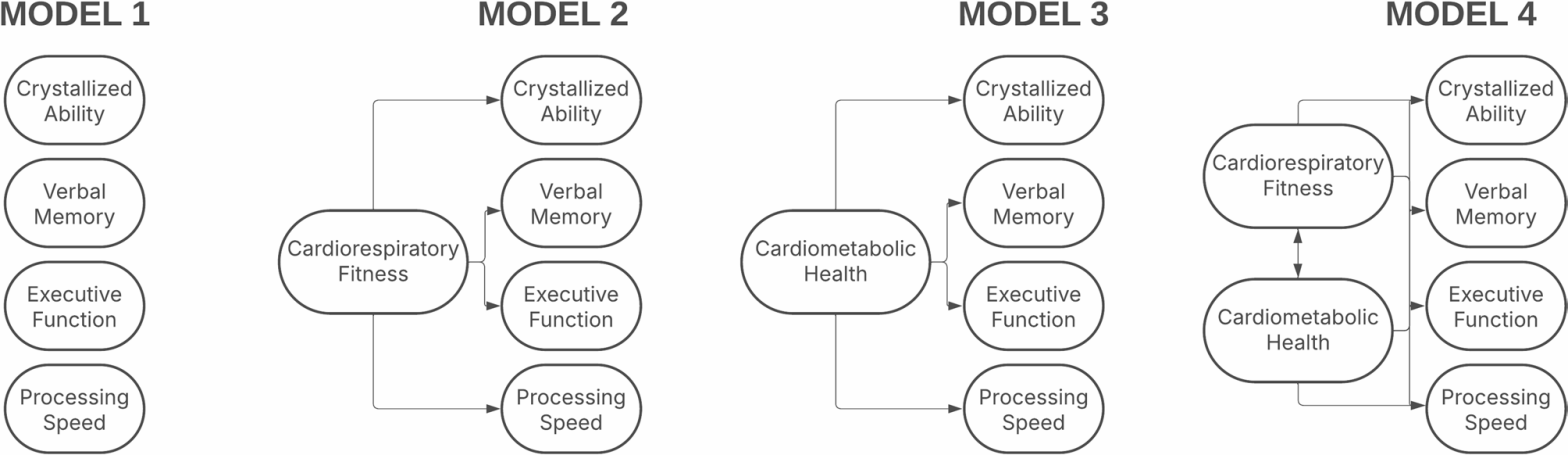
Schematic structure of conceptual models. Model 1: Four cognitive ability factors; Model 2: Direct associations between Cardiorespiratory Fitness factor and cognitive ability factors; Model 3: Direct associations between Cardiometabolic Health factor and cognitive ability factors; Model 4: Direct association between Cardiorespiratory Fitness and Cardiometabolic Health factors, and their unique associations with cognitive ability factors when controlling for shared variance. Created in Lucid (lucid.co).

Using structural equation modeling (SEM), we first created a model of cognitive ability with four latent cognitive factors covering the above domains (Fig. 1, Model 1). Models 2 and 3 assessed the unique associations between these cognitive domains and cardiorespiratory fitness and cardiometabolic health, respectively. Model 4 included both measures of cardiovascular health in order to examine whether the pattern of unique associations found in Models 2 and 3 remain when controlling for their shared variance.

## Methods

### Ethics

This study uses Phase 1 data from the longitudinal ACTIVate Study^42^ collected between August 2020 and February 2022 at the University of South Australia and the University of Newcastle. All experimental protocols used in the current study were carried out in accordance with the Declaration of Helsinki and with guidelines approved by the University of South Australia and the University of Newcastle. Ethics approval was obtained from the University of South Australia and registered with the University of Newcastle Human Research Ethics Committee (H-2019-0421). ACTIVate was registered with the Australian New Zealand Clinical Trials Registry (ACTRN12619001659190) on November 27, 2019. Full, written, informed consent was obtained from all participants.

### Participants

This study analyzed data from all 426 participants who met the eligibility criteria for the ACTIVate Study. The ACTIVate Study was designed to investigate how lifestyle patterns influence brain function and cognitive health during a critical period of aging, i.e., when subtle physiological and cognitive changes may begin to emerge but before the onset of marked decline or increased dementia risk^42^. Eligibility criteria included age between 60 and 70 years, fluency in English, and the absence of any exclusionary medical conditions. Participants were not invited to the study if they had color-blindness, a clinical diagnosis of a major neurological or psychiatric disorder, intellectual disability or major physical disability, or mild cognitive impairment or dementia (i.e., a score < 13/22 on the Montreal Cognitive Assessment – Blind/Telephone version; MoCA-B^43^), as assessed via telephone interview. Participants were primarily White Anglo-Saxon, with 79% born in Australia.

**Fig. 2:**
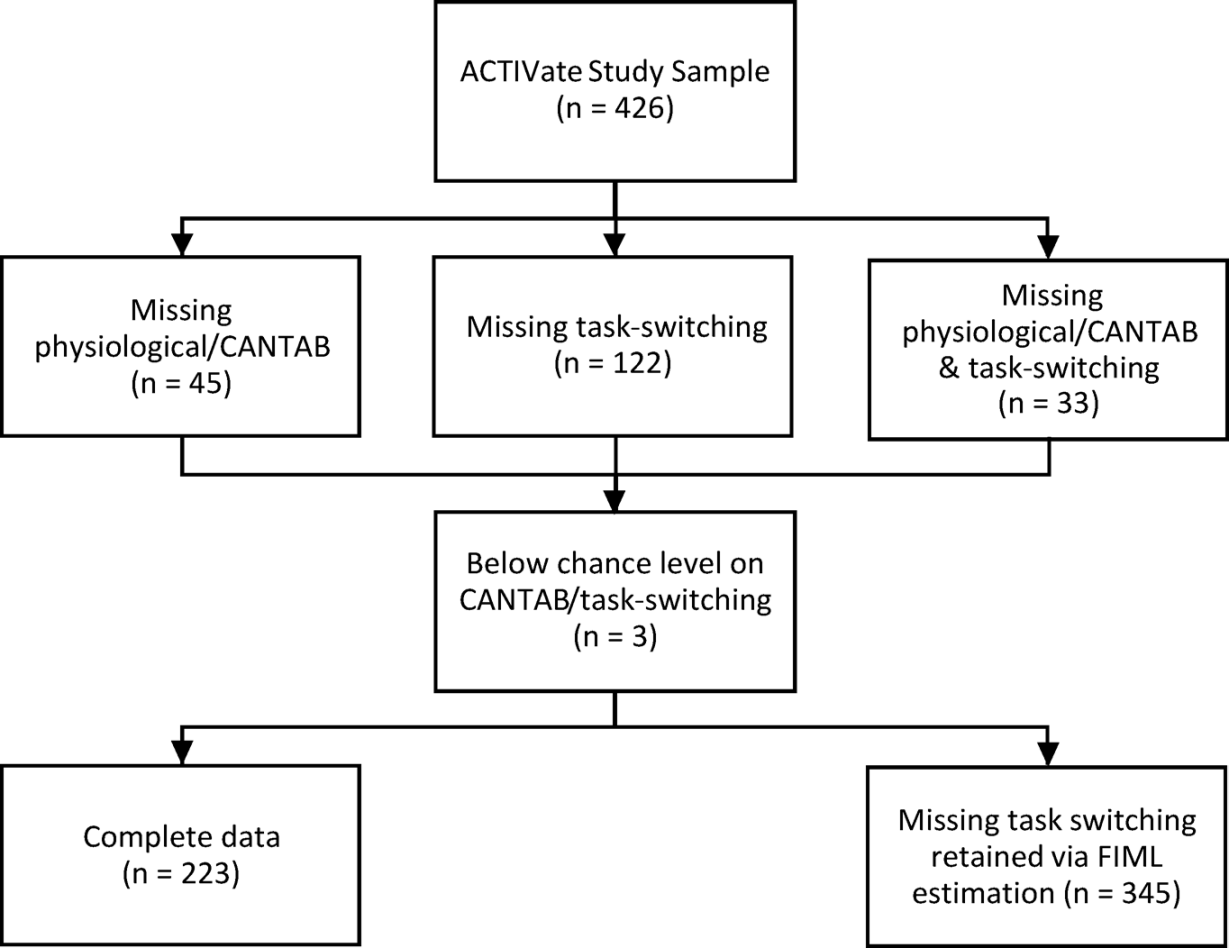
Flow diagram of participant inclusion and exclusion for the current study.

Figure 2 presents a flow diagram of participant inclusion and exclusion for the current study. Of the 426 participants in the ACTIVate cohort, 45 had missing data on physiological measures and/or other neuropsychological tasks, 122 did not complete the task-switching paradigm, and 33 had missing data on both, resulting in a complete data set for this study of *n* = 226. Of these, three participants were excluded because they performed below chance level on the CANTAB Multitasking Test or task-switching paradigm, resulting in a complete data set of 223. The 122 participants without task-switching data who had physiological/neuropsychological data were included in the modelling using Full Information Maximum Likelihood (FIML) procedures to increase statistical power (see below). Consequently, the analyses reported here are derived from a sample of 345 participants.

To verify whether missing data affected the pattern of results, we also ran the analyses using the smaller sample with complete data (*n* = 223). The pattern of findings remained largely unchanged (see supplementary analyses:https://osf.io/pu8st/).

### Demographic measures and cognitive tasks

Demographic, past and current clinical history were collected during a brief interview. Socioeconomic status (SES) was quantified using postcode data to derive the Index of Relative Socio-economic Advantage and Disadvantage (IRSAD) from Australian Census data. IRSAD measures a suburb’s socio-economic advantage (e.g., high income, skilled occupations) and disadvantage (e.g., low income, unskilled occupations), with higher scores indicating areas with greater relative advantage^44^. However, it does not quantify individual differences amongst residents within a suburb. Therefore, as IRSAD values showed limited variability in this relatively homogeneous, community-dwelling cohort, they were used as background descriptors of the cohort (see Table 2) and not included as covariates in the analyses.

Overall cognitive functioning was screened using the Addenbrooke’s Cognitive Examination III (ACE-III), a brief cognitive screening tool encompassing five domains (Attention, Memory, Fluency, Language, and Visuospatial Ability)^45^. Because all participants scored well above the clinical cutoff (score ≥ 82/100; see Table 2) and the measure is designed for diagnostic rather than continuous assessment of cognitive performance, ACE-III scores were not included in the analyses.

Cognitive measures included in the models were derived from the Multitasking Test, Reaction Time task and Verbal Recognition Memory task of the Cambridge Neuropsychological Test Automated Battery (CANTAB, Cambridge Cognition, 2023)^46^, the Oral Reading Recognition Test and Picture Vocabulary Test of the National Institute of Health Toolbox (NIH) Cognition Battery (NIHTB-Cog)^47,48^ and the cued-trials task-switching paradigm^49^. The specific measures selected from each of the above tests are outlined in Table 1.

The cued-trials task-switching paradigm (TSWT)^49^ was adapted from Whitson et al.^50^ and followed the same experimental parameters and data preprocessing approach. Briefly, participants were trained on two simple classification tasks (i.e., *is number odd or even*,* is the letter a vowel or consonant*) to establish cue-target and target-response mappings. Each trial consisted of a cue (i.e., colored cross) that reliably indicated which task to perform on the subsequent target (C-T interval = 1000 msec). Targets consisted of two characters. One was from the cued task (e.g., a letter, if the letter task was cued). The other character was either a non-alphanumeric character (*neutral target*, e.g.,* % or &* that was not mapped to any response) or an exemplar from the uncued task that was mapped to an incongruent response (*incongruent target*, e.g., if the letter task was cued, and the letter was mapped to a left-hand response, the number was mapped to a right-hand response). Participants completed the letter and number tasks either alone (single-task block) or in a random alternating sequence (mixed-task block). Trials associated with incorrect responses or with reaction times (RT) that were implausibly fast (< 200 ms) or excessively slow (exceeded mean RT + 3 × *SD* of RTs for that condition) were labelled as invalid and excluded from all analyses. For the remaining valid trials, median RT and average error rate were estimated in single-task and mixed-task blocks, and for all incongruent and all neutral trials (see Table 1).

**Table 1:** Overview of the cognitive variables.

Measurement tool	Variable name	Operationalisation
NIH Toolbox Cognitive Battery^a^: **Oral Reading Recognition Test (ORRT)** Expressive language	Oral reading recognition	The proportion of correctly pronounced letters and words.
NIH Toolbox Cognitive Battery^a^: **Picture Vocabulary Test (PVT)** Receptive language	Picture vocabulary recognition	The proportion of correctly selected pictures that best match the orally presented words.
CANTAB^b^: **Reaction Time (RTI)** Psychomotor speed	Simple RT	The sum of median reaction time (i.e., time from stimulus appearance to initiation of the response) and median movement time (i.e., time from response initiation to response completion).
	Choice RT	The sum of median reaction time (i.e., time from stimulus appearance to initiation of the response) and median movement time (i.e., time from response initiation to response completion).
CANTAB^b^: **Verbal Recognition Memory (VRM)** Verbal episodic memory	Immediate free recall	The total number of correctly recalled target words during the immediate free recall phase.
	Immediate recognition	The total number of correctly recognised target words and correctly rejected distractor words in the immediate recognition phase.
	Delayed recognition	The total number of correctly recognised target words and correctly rejected distractor words in the delayed recognition phase.
CANTAB^b^: **Multitasking Test (MTT)** Task-set shifting and interference control	MTT multitasking cost (IES)	The difference in median reaction time between multitasking blocks (random sequence alternating between two rules: respond to the side on which the arrow appears vs. the direction the arrow points) and single-task blocks (respond to either side or direction across all trials).
	MTT congruency cost (IES)	The difference in median reaction time between incongruent (e.g., arrow in left side but points right) and congruent (e.g., arrow in left side and points left) trials, averaged across single and multitasking blocks.
**Task-switching paradigm (TSWT)** ^**c**^ Task-set shifting and interference control	TSWT multitasking cost (IES)	The difference in mean reaction time between mixed-task blocks (randomly alternating between letter and number classification tasks) and single-task blocks (repeating the same task across all trials), averaged across incongruent and neutral trials. Often referred to as General Switch Cost.
	TSWT congruency cost (IES)	The difference in mean reaction time between incongruent trials (i.e., target contains features from both cued and uncued tasks that are mapped to different responses) and neutral trials (i.e., irrelevant target feature is not mapped to any response) averaged across single-task and mixed-task blocks.

Note. ^a^NIH Toolbox^®^ for Assessment of Neurological and Behavioral Function Administrator’s Manual (2006–2020); ^b^ Cambridge Cognition Ltd. CANTABeclipse Test Administration Guide (2012); ^c^ Whitson et al.^50^.

### Physiological and fitness measures

Physiological measures (i.e., systolic and diastolic blood pressure, resting heart rate, and fasted blood-based metabolic markers) and anthropometric measures (i.e., BMI calculated from height and weight) were obtained using standard procedures^42^.

Fasted blood samples were obtained via venipuncture, centrifuged at 4000 rpm for 10 min, and the plasma was aliquoted and stored at − 80 ◦C. Prior to the analysis, samples were thawed on ice, vortexed, and centrifuged (10 K rpm for 2 min). Total cholesterol, triglycerides, HDL and glucose were analyzed using a KONELAB 20XTi auto-analyzer with specific Thermo Fisher reagents. Low-density lipoprotein (LDL) was calculated using the equation LDL = (total cholesterol − HDL) − (trig/2.17).

Total time per day in moderate-to-vigorous physical activity (MVPA) was quantified from accelerometry data recorded over 7 consecutive days using a triaxial accelerometer (Axivity AX3) on the non-dominant wrist. Data were pre-processed using Open Movement GUI software (OmGUI) and analyzed with a custom MATLAB toolbox (COBRA; MATLAB R2018b)^51^.

Hypertension and hypercholesterolemia were defined using Australian guidelines^52^ and self-report. Hypertension was considered present if the participant had an average systolic blood pressure reading over > 140 mmHg, reported having hypertension or using blood pressure medication. Hypercholesterolaemia was considered present if they had a total cholesterol reading over 5.5 mmol/L (213 mg/dL), reported having, or having received a diagnosis of high cholesterol by a health professional or reported using statin medication.

### Data preparation

The SEM analysis included socio-demographic variables (age, sex, years of completed education), hemodynamic variables (systolic and diastolic blood pressure in mmHg, resting heart rate as beats per minute), and blood metabolic variables (HDL, glucose, and triglycerides), as well as BMI as a proxy for body fat and MVPA as an indicator of physical activity level. It also included eleven cognitive variables listed in Table 1.

For the CANTAB Multitasking Test and the task-switching paradigm, RT multitasking cost and congruency cost scores were adjusted for accuracy (i.e., RT/*p*(correct)), yielding the inverse efficiency score (IES)^53^. IES captures both the speed and accuracy of responses and has a more normal distribution than raw RT-based measures. This is particularly important when testing older individuals, who often show a speed-accuracy trade-off^54^.

All variables were adjusted, so that higher values for all variables indicate better condition/performance. To achieve this, values for blood pressure, heart rate, glucose, triglycerides, BMI, simple and choice RT, multitasking and congruency costs were reversed. All variables were screened for their distribution and subsequently scaled (i.e., standardized to have *M* = 0 and *SD* = 1).

### Structural equation modeling (SEM)

SEM was employed to characterize the structure of cognitive domains. In subsequent analyses, the two cardiovascular latent variables were introduced sequentially—first separately, and then together—as predictors of these cognitive domains (Fig. 1).

FIML was used to account for missing task-switching data for 122 participants in order to maximize sample size. The variance of the latent variables was fixed to 1. Model fit was assessed using standard indices, including the root-mean-square error of approximation (RMSEA), standardised root-mean-square residual (SRMR), and comparative fit index (CFI). Conventionally, a good fit is indicated by RMSEA < 0.05, SRMR < 0.08 and CFI > 0.95^55^.

The data were analyzed and visualized in R (version 4.1.0)^56^, using the following packages: “tidyverse”^57^, “psych”^58^, “Hmisc”^59^, “lavaan”^60^. The code is available in the project repository (https://osf.io/pu8st/). Requests for data access can be made through the ACTIVate Study^42^.

#### Model 1: Cognitive Ability Model

 We tested the feasibility of a Cognitive Ability Model with four cognitive factors that vary in the degree to which they are impacted by age. The *Crystallized Ability factor* loaded on measures associated with semantic knowledge (Oral Reading Recognition and Picture Vocabulary Recognition from the NIH-Toolbox-Cb), and years of education, which is often a proxy for cognitive reserve^61^. The *Verbal Memory factor* loaded on Free Recall, Immediate and Delayed Recognition scores from the Verbal Recognition Memory test. The *Executive Function factor* loaded on Multitasking and Congruency Costs from both the Multitasking Test and the task-switching paradigm. The *Processing Speed factor* loaded on Simple and Choice RT.

All factors were allowed to correlate with each other. The residual errors of variables derived from the same tasks were correlated to account for shared task-specific variance (i.e., MTT Congruency Cost with MTT Multitasking Cost, TSWT Congruency Cost with TSWT Multitasking Cost). By allowing for correlated residuals, the model better reflects the underlying structure of the data and reduces the risk of misinterpreting task-specific or methodological influences as true relationships between variables. Since the Processing Speed factor consisted of only two variables, their loadings were constrained to be equal. This constraint ensures that both indicators equally represent the “Processing Speed” construct and addresses the potential issue of under-identification when a latent variable has few but highly correlated indicators^62^.

####  Model 2: Cardiorespiratory Fitness Model

Consistent with validated non-exercise fitness estimation equations (i.e., Jurca score)^21,63^, the *Cardiorespiratory Fitness latent variable* included resting heart rate, MVPA and BMI. We deliberately replicated the variable selection used in established non-exercise fitness equations to ensure our latent fitness construct aligns with proven methodological approaches in cardiorespiratory fitness assessment. Model 2 assessed associations between the Cardiorespiratory Fitness factor and the four cognitive domains in the Cognitive Ability Model.

####  Model 3: Cardiometabolic Health Model

Based on established algorithms for assessing general risk of cardiovascular diseases (e.g., FRS (Framingham Risk Score), CAIDE (Cardiovascular Risk Factors, Aging, and Incidence of Dementia))^6,64^, the *Cardiometabolic Health latent variable* was derived using key measures of cardiometabolic condition: systolic and diastolic blood pressure, glucose, HDL, triglycerides and BMI. Although certain behavioral or clinical factors (e.g., smoking, diabetes, use of antihypertensive or lipid-lowering medication) are components of clinical risk formulas, these variables were not included in the latent construct. This is because their physiological consequences (e.g., altered blood pressure, glucose metabolism, lipid levels) are already represented in the continuous biomarkers that form the latent variable. Therefore, including such categorical variables would introduce redundancy and conflate behavioral or diagnostic status with underlying physiological function. Model 3 assessed associations between the Cardiometabolic Health factor and cognitive abilities in the Cognitive Ability Model. The residual errors of variables derived from the same measurements were correlated (i.e., systolic with diastolic blood pressure; HDL with triglycerides).

#### Model 4: Cardiovascular Health Model

The final Cardiovascular Health Model included Cardiorespiratory Fitness and Cardiometabolic Health as latent variables. These were modelled concurrently to (1) statistically adjust for their covariance and (2) isolate their independent associations with the four cognitive domains.

When selecting indicators for a latent variable in SEM, it is important to ensure that the factor captures the intended theoretical construct rather than being disproportionately influenced by a subset of indicators. While our dataset includes an additional measure of body composition (i.e., waist-hip ratio) and other lipid profile markers (i.e., total cholesterol and LDL), we deliberately excluded these variables from modeling. Including them would result in the latent variables being overly dominated by highly correlated indicators shifting their meaning primarily toward body composition and lipid profile, respectively. Instead, to maintain conceptual clarity in the current analyses, we selected variables that are more widely used in the literature (i.e., BMI instead of waist-hip ratio)^64,65^ and considered more reliable indicators of lipid metabolism (i.e., HDL instead of total cholesterol or LDL)^66,67^. Importantly, the pattern of results remains virtually unchanged when alternative indicators are used. For instance, we estimated alternative cardiometabolic factors by replacing HDL with total cholesterol or LDL in Model 2. Both were highly correlated with the original cardiometabolic factor (*r* = 0.99 and *r* = 0.98, respectively). This indicates that our choice does not bias the findings but rather enhances the interpretability of the latent variables.

Residual errors of variables sharing physiological measurement pathways were permitted to covary (e.g., systolic/diastolic blood pressure; HDL/triglycerides) to account for shared biological variance beyond the factors.

To remain consistent with established cardiovascular health frameworks, BMI was retained as an indicator of *both* latent variables, reflecting its dual association with cardiorespiratory capacity (e.g., via aerobic efficiency) and cardiometabolic risk (e.g., via adiposity-related pathways). To evaluate whether this shared indicator introduced confounding, we conducted a sensitivity analysis (Model 4, Fig. 6, Panels C and D) excluding BMI from both latent constructs.

### Controlling for age

As the cohort was selected to have a narrow age range (i.e., 10 years), we did not anticipate significant age-related influences. To confirm this, we re-fitted Models 1–3, allowing age to correlate with the Cardiorespiratory Fitness and Cardiometabolic Health factors and predict cognitive factors. These models showed substantially poorer fit and, importantly, age did not correlate with either latent cardiovascular variable or account for the observed relationships (see: https://osf.io/pu8st/). Therefore, we report SEM analyses without age.

### Controlling for sex

We also examined whether the pattern of relationships between cardiovascular health patterns and cognitive outcomes varied across sex. Factor values for cardiovascular and cognitive variables were extracted from Model 4. First, independent t-tests were conducted to evaluate sex group differences in Cardiorespiratory Fitness and Cardiometabolic Health. Next, we used linear regression to investigate whether sex moderated the relationships between cardiovascular health dimensions and cognitive factors. Specifically, we fitted models in which the cardiovascular predictor (Cardiorespiratory Fitness or Cardiometabolic Health) was included alongside one of the aforementioned variables and their interaction term. These models were tested separately for each cognitive factor: Crystallized Ability, Verbal Memory, Executive Function, and Processing Speed.

## Results

### Cohort characteristics

Table 2 presents summary statistics of socio-demographic, physiological, and cognitive measures for the entire sample of the cohort of 345 older adults and separated by sex. The mean age was 65 yrs with a range of 60–70 years, by design. On average, the cohort was highly functioning (mean ACE-III = 95)^68,69^, with females performing slightly better than males. There were no other sex differences in basic demographics. On average, the cohort was highly educated, with most participants having completed post-secondary education. The mean IRSAD score of 1023 indicated a moderate socio-economic advantage, above the Australian mean of 1003 and the New South Wales mean of 1016.

Participants were highly active (average of 1.5 h/day MVPA), with higher activity in males (Table 2). Yet, BMI was in the middle of the overweight range for both sexes. On average, measures of cardiovascular health were within the healthy range^52^. Males had significantly higher systolic and diastolic BP, but lower resting heart rate and lower HDL than females (Table 2). As shown in Table 3, 53.3% of individuals did not meet the criteria for a hypertension diagnosis. Of the remaining 46.7% with hypertension, 24.1% were on one or more anti-hypertensive medications. Overall, 67.5% of individuals had hypercholesterolaemia and, of these, 16.2% were on medication to manage their cholesterol. Cognitive performance in the present sample falls within the range typically observed in healthy older adults aged 60 years and above (e.g.,^70–72^). RT measures were consistent with previous literature in this age group (e.g.,^73,74^).

### SEM results

All variables exhibited substantial variability, as indicated by their standard deviations. Most variables were normally distributed, except for three metabolic variables (HDL, glucose, and triglycerides) which were normalized using log10 transformation.

Reliability of blood pressure and heart rate (three measurement points), BMI (two measurement points) were evaluated using the intra-class correlation coefficient. We did not assess the reliability of MVPA measures taken over seven days, as habitual activity varies across days and between weekdays and weekends. The reliability of measures derived from the task-switching paradigm was determined using split-half correlations and adjusted with the Spearman-Brown prophecy formula. As shown in Table 4, all measures were highly reliable. Metabolic variables (i.e., glucose, HDL, triglycerides) had a single measurement point so reliability could not be established. For CANTAB and NIH measures, we did not have access to raw data required for independent reliability calculations. Previous studies have reported adequate reliability for the NIH Toolbox^75^ and CANTAB^76,77^ tasks.

**Table 2:** Mean (standard deviation) for demographic, physiological, and cognitive measures.

Variable	Full sample *n* = 345	Females *n* = 239	Males *n* = 106	t-test (two-sided)
Age (yrs)	65.46 (2.97)	65.31 (2.84)	65.80 (3.22)	*t*(343) = 1.41, *p* = 0.16
Education (yrs)	16.64 (3.24)	16.48 (3.31)	16.98 (3.06)	*t*(343) = 1.32, *p* = 0.19
ACE-III (dementia considered < 82/100)	95 (4)	95 (3)	94 (4)	***t(*** **343) = -2.20**, ***p*** **= 0.03**
SES (New South Wales mean = 1016.0)	1023.3 (71.05)	1021.40 (70.80)	1027.58 (71.78)	*t*(320) = 0.72, *p* = 0.47
MVPA (recommended 30 min/day)	88.69 (46.85)	82.34 (42.62)	103.02 (52.68)	***t*** **(343) = 3.86**, ***p*** **< 0.001**
BMI (obese > 30 kg/m^2^)	27.00 (5.20)	26.84 (5.49)	27.36 (4.50)	*t*(343) = 0.86, *p* = 0.39
Glucose (normal < 150 mg/dL)	92.07 (12.50)	91.37 (12.91)	93.65 (11.43)	*t*(343) = 1.56, *p* = 0.12
HDL cholesterol (normal > 60 mg/dL)	68.43 (20.42)	73.30 (19.98)	57.46 (16.92)	***t*** **(343) = -7.11**, ***p*** **< 0.001**
Triglycerides (normal < 150 mg/dL)	101.93 (49.48)	100.29 (46.97)	105.65 (54.80)	*t*(343) = 0.93, *p* = 0.35
Resting heart rate (normal 60–100 bpm)	66.49 (10.13)	67.92 (9.50)	63.27 (10.80)	***t*** **(343) = -4.02**, ***p*** **< 0.001**
Brachial systolic BP (normal < 140 mmHg)	134.36 (17.78)	130.49 (16.96)	143.09 (16.51)	***t*** **(343) = 6.42**, ***p*** **< 0.001**
Brachial diastolic BP (normal < 80 mmHg)	81.04 (10.14)	80.23 (9.68)	82.86 (10.95)	***t*** **(343) = 2.34**, ***p*** **= 0.03**
NIH toolbox reading recognition	119.00 (13.43)	118.87 (12.58)	119.31 (15.24)	*t*(343) = 0.28, *p* = 0.78
NIH toolbox picture vocabulary	113.13 (9.83)	113.38 (9.95)	112.57 (9.59)	*t*(343) = -0.71, *p* = 0.48
VRM immediate recognition	32.38 (2.53)	32.36 (2.59)	32.44 (2.39)	*t*(343) = 0.30, *p* = 0.77
VRM delayed recognition	31.51 (2.79)	31.65 (2.71)	31.19 (2.97)	*t*(343) = -1.43, *p* = 0.16
VRM free recall	5.88 (2.21)	6.04 (2.23)	5.52 (2.14)	*t*(343) = -2.02, *p* = 0.05
MTT multitasking cost	299.90 (184.56)	313.85 (193.22)	268.42 (159.79)	***t*** **(343) = -2.12**, ***p*** **= 0.04**
MTT congruency cost	137.76 (104.23)	146.16 (110.94)	118.82 (84.67)	***t*** **(343) = -2.26**, ***p*** **= 0.02**
TSWT multitasking cost	170.17 (151.28)	170.95 (137.78)	168.67 (175.44)	*t*(221) = -0.11, *p* = 0.92
TSWT congruency cost	154.77 (82.13)	164.69 (81.54)	135.85 (80.36)	***t*** **(221) = -2.54**, ***p*** **= 0.01**
Simple RT (msec)	571.81 (78.75)	572.44 (77.43)	570.40 (82.00)	*t*(343) = -0.22, *p* = 0.83
Choice RT (msec)	660.58 (78.78)	661.45 (80.11)	658.63 (76.03)	*t*(343) = -0.31, *p* = 0.76

Addenbrooke’s Cognitive Examination III: A measure of global cognitive function, scored out of 100; SES: Socio-economic status, derived from the Index of Relative Socio-economic Advantage and Disadvantage^44^ ; MVPA: Moderate-to-Vigorous Physical Activity, measured in minutes per day. For detailed descriptions of CANTAB, NIH and task switching variables, refer to Table 1. Significant differences (*p* < 0.05) between females and males are bolded. Due to missing data on specific measures, sample sizes varied across comparisons—*n* = 223 for TSWT measures and *n* = 345 for most variables—as indicated by the corresponding degrees of freedom reported in the “*t*-test (two-sided)” column.

**Table 3:** Presence of hypertension, hypercholesterolemia and medication status by sex.

	Hypertension			Hypercholesterolemia		
	Not present	Present		Not present	Present	
		Not Medicated	Medicated		Not Medicated	Medicated
Overall	53.3% (*n* = 184)	22.6% (*n* = 78)	24.1% (*n* = 83)	32.5% (*n* = 112)	51.3% (*n* = 177)	16.2% (*n* = 56)
Female	60.7% (*n* = 145)	17.6% (*n* = 42)	21.8% (*n* = 52)	24.3% (*n* = 58)	60.3% (*n* = 144)	15.5% (*n* = 37)
Male	36.8% (*n* = 39)	34.0% (*n* = 36)	29.3% (*n* = 31)	50.9% (*n* = 54)	31.1% (*n* = 33)	17.9% (*n* = 19)

The presence of cardiometabolic risk factors was determined using physiological measures (e.g., blood tests, blood pressure) that exceeded Australian guideline thresholds (e.g., total cholesterol > 5.5 mmol/L, systolic BP > 140 mmHg, blood glucose > 7.0 mmol/L)^52^. Participants were also classified as having a risk factor if they reported a diagnosis from a qualified professional or were on related medication.

**Table 4:** Reliability of measurement tools.

Variable	Reliability (*r*)
Systolic BP	0.95
Diastolic BP	0.94
Resting HR	0.97
BMI	1.00
TSWT multitasking	0.99
TSWT congruency	0.99

Intra-class correlation for systolic blood pressure, diastolic blood pressure, heart rate and BMI; odd-even Pearson’s *r* with Spearman-Brown adjustment for TSWT Multitasking Cost and TSWT Congruency Cost. *N* = 223 for the TSWT Multitasking Cost and TSWT Congruency Cost correlations; *N* = 345 for the remaining.

Pearson correlations between key demographic and cognitive variables are reported in Table 5. As expected, variables related to cognitive abilities showed moderate-to-strong intercorrelations. Despite a restricted age range (60–70 years), measures contributing to the Processing Speed and Executive Function factors showed weak but significant correlations with age.

A sensitivity analysis indicated that, with *n* = 345 and α = 0.05, the study had 80% power to detect standardized path coefficients of approximately *β* = 0.15 and over 95% power for *β* = 0.20. All SEM models demonstrated satisfactory fit, as indicated by the fit indices summarized in Table 6 and described in detail below.

It is important to note that all variables contributing to the models were coded such that higher scores reflect better cognitive performance or more favorable physiological condition.

**Table 5:** Correlations between age and cognitive variables (upper panel), age and physiological variables (middle panel), cognitive and physiological variables (lower panel).

A. Cognitive Variables	2	3	4	5	6	7	8	9	10	11	12	13
	Educ	RRec	PVoc	ImmR	DelR	FreeR	MTT-M^	MTT-C^	TSWT-M^	TSWT-C^	sRT^	cRT^
1. Age	-0.02	-0.01	-0.02	-0.02	-0.04	**-0.12**	-0.08	**-0.21**	**-0.19**	**-0.19**	**-0.12**	**-0.16**
2. Education	–	**0.27**	**0.24**	0.04	0.09	0.09	0.03	**0.16**	**0.13**	**0.03**	-0.02	-0.03
3. Read recognition		–	**0.47**	0.09	**0.23**	**0.25**	**0.15**	**0.19**	**0.24**	**0.17**	-0.08	-0.10
4. Pict vocabulary			–	0.10	**0.14**	**0.19**	**0.13**	**0.15**	**0.25**	**0.12**	-0.06	-0.05
5. Imm recognition				–	**0.62**	**0.40**	**0.12**	0.05	0.06	0.05	0.06	0.05
6. Delay recognition					–	**0.46**	**0.16**	0.10	0.06	0.10	0.05	0.02
7. Free recall						–	0.00	0.06	0.10	**0.16**	0.05	0.02
8. MTT multitasking^							–	**0.25**	**0.22**	**0.22**	**-0.12**	**-0.13**
9. MTT congruency^								–	**0.18**	**0.28**	0.02	0.02
10. TSWT multitasking^									–	**0.64**	0.04	0.05
11. TSWT congruency^										–	0.09	**0.14**
12 Simple RT^											–	**0.84**
13. Choice RT^												–

B. Physiological variables	2	3	4	5	6	7	8	9				
	MVPA	BMI^	Gluc^	HDL	Trigl^	rHR^	sBP^	bBP^				
1. Age	-0.04	-0.02	-0.05	-0.01	**0.04**	-0.03	**-0.14**	**-0.01**				
2. MVPA (mins/week)	–	**0.36**	0.04	**0.20**	**0.26**	**0.21**	0.02	0.02				
3. BMI (kg/m^**2**^**)^**		–	**0.32**	**0.42**	**0.38**	**0.18**	**0.18**	**0.21**				
4. Glucose (mg/dl)^			–	**0.16**	**0.23**	**0.12**	0.02	**0.21**				
5. HDL Cholesterol (mg/dl)				–	**0.48**	0.04	**0.12**	0.06				
6. Triglycerides (mg/dl)^					–	**0.14**	0.09	**0.17**				
7. Resting Heart Rate (bpm)^						–	-0.09	**0.18**				
8. Brachial Systolic BP (mmHg)^							–	**0.62**				
9. Brachial Diastolic BP (mmHg)^								–				

C. Cognitive and physiological	MVPA	BMI^	Gluc^	HDL	Trigl^	rHR^	sBP^	dBP^				
Read recognition	-0.07	0.08	**0.13**	0.05	0.04	0.04	0.07	**0.16**				
Pict vocabulary	-0.00	0.08	-0.02	0305	0.00	-0.03	**0.14**	0.05				
Imm recognition	0.05	0.02	0.00	-0.00	0.07	-0.04	-0.01	**0.11**				
Delay recognition	0.01	0.08	**0.11**	0.04	0.07	-0.03	-0.04	0.09				
Free recall	-0.00	0.09	0.07	0.03	0.05	-0.05	0.04	0.10				
MTT multitasking ^	-0.04	-0.05	-0.03	0.05	0.00	**-0.12**	0.00	-0.01				
MTT congruency ^	-0.03	-0.05	-0.11	0.07	-0.10	**-0.12**	-0.02	-0.04				
TSWT multitasking ^	**-0.13**	-0.07	-0.04	-0.05	-0.07	**-0.13**	-0.03	0.03				
TSWT congruency ^	**-0.24**	-0.13	-0.07	-0.07	**-0.15**	**-0.27**	0.03	0.05				
Simple RT ^	-0.12	-0.08	0.04	-0.01	-0.04	-0.08	0.00	-0.04				
Choice RT ^	**-0.14**	**-0.13**	-0.00	-0.07	-**0.12**	**-0.12**	0.02	-0.01				

For detailed descriptions of CANTAB, NIH and task switching variables, refer to Table 1. Variables marked with ^ were reverse-coded so that higher values indicate better physiological condition or cognitive performance. *p* < 0.05 bolded.

**Table 6:** Goodness of fit statistics for the structural equation models.

Model	CFI	RMSEA [90% CI]	SRMR
Cognitive Ability	0.981	0.034 [0.010, 0.053]	0.041
Cardiorespiratory Fitness	0.974	0.033 [0.014, 0.047]	0.044
Cardiometabolic Health	0.966	0.034 [0.021, 0.046]	0.043
Cardiovascular Health	0.959	0.035 [0.024, 0.048]	0.045
Cardiovascular Health (no BMI)	0.963	0.034 [0.022, 0.045]	0.045

CFI, Comparative fit index; RMSEA, Root mean square error of approximation; SRMR, Standardized root mean squared residual.

### Model 1: Cognitive Ability (Fig. 3)

All factors significantly loaded onto their respective variables with coefficients varying between 0.36 and 0.92. For the Crystallized Ability factor, the two semantic knowledge tasks had higher loadings than years of education. For the Verbal Memory factor, both recognition scores had higher loading than immediate recall scores. For the Executive Function factor, the four cost variables had roughly equal loadings.

Crystallized Ability, Verbal Memory and Executive Function factors correlated moderately to strongly with each other (*r* = 0.25–0.54, *p* < 0.05), with higher crystallized ability associated with higher executive function and better verbal memory. The Processing Speed factor was not significantly associated with any factor, consistent with representing psychomotor speed rather than a cognitive ability^78^.

These relationships remained highly consistent when the cognitive factors were included in Models 2–4 (see Figs. 4A, 5A and 6A).

**Fig. 3:**
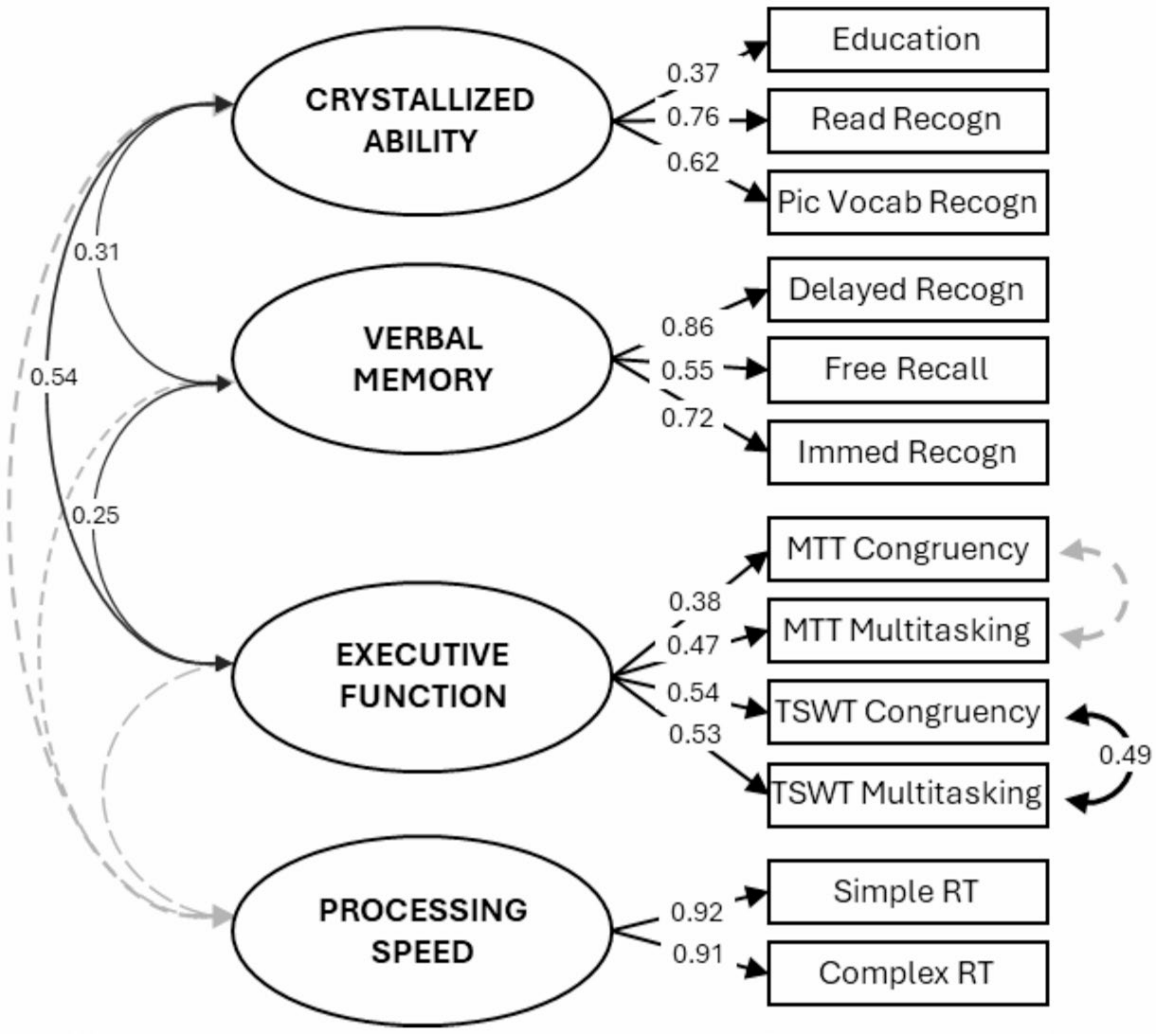
Model 1: Cognitive Ability Model. Standardized covariance coefficients (in 2-way arrows) show associations between cognitive factors (ovals). Standardized factor loadings of factors on manifest variables (rectangles) are shown in 1-way arrows. Simple and Complex RT, as well as both Congruency and Multitasking variables were reverse scored to ensure that, for both latent and manifest variables, higher scores consistently reflect better performance or more favorable outcomes (e.g., higher education, better recall, lower task cost, faster reaction times). Solid black lines indicate *p* < 0.05; dashed grey lines indicate *p* ≥ 0.05. For cognitive variable names, see Table 1. For confidence intervals, see: https://osf.io/pu8st/.

### Model 2: Cardiorespiratory Fitness (Fig. 4)

The Cardiorespiratory Fitness factor showed significant loadings on BMI, resting heart rate and MVPA (loadings = 0.38–0.63), with MVPA having the highest loading. This factor was positively associated with Executive Function (*β* = 0.36) and Processing Speed (*β* = 0.23), indicating that individuals with higher cardiorespiratory fitness tended to perform better on executive function tasks and had faster processing speed. In contrast, there were no significant associations between the Cardiorespiratory Fitness factor and either Crystallized Ability or Verbal Memory (*β* = 0.03 and 0.05, respectively; both *p* > 0.05).

### Model 3: Cardiometabolic Health (Fig. 5)

The Cardiometabolic factor significantly loaded on systolic and diastolic blood pressure, glucose, HDL, triglycerides and BMI, with weakest loadings for systolic blood pressure (*β* = 0.21) and highest for BMI (*β* = 0.77).

The Cardiometabolic Health factor was weakly but significantly associated with Crystallized Ability and Verbal Memory (*β* = 0.16 and 0.13, respectively), such that higher cardiometabolic health corresponded to higher scores in these cognitive domains. Cardiometabolic Health showed similar loadings for Executive Function and Processing Speed (*β* = 0.17 and 0.13), but neither was statistically significant (both *p* > 0.05).

**Fig. 4:**
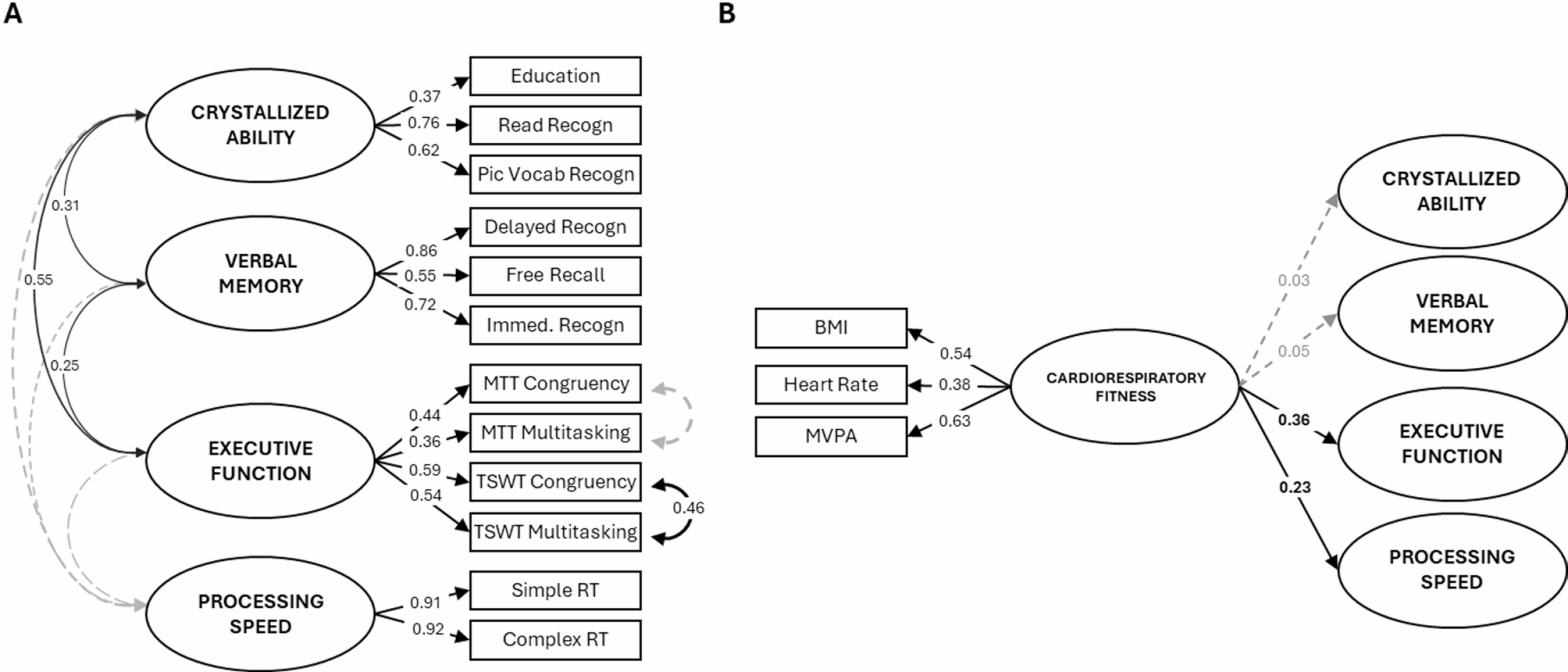
Model 2: Cardiorespiratory Fitness Model. Panel (**A**) shows factor loadings for cognitive factors and their covariances as estimated when examining the association between the Cardiorespiratory Fitness factor and each cognitive factor. Note that there is little difference in loadings compared to Model 1. Panel **B** shows standardized factor loadings of the Cardiorespiratory Fitness factor on manifest variables (*BMI: body mass index; MVPA: moderate-to-vigorous physical activity*) and standardized path coefficients from the Cardiorespiratory Fitness factor to each cognitive factor. Simple and Complex RT, as well as both Congruency and Multitasking variables were reverse scored to ensure that, for both latent and manifest variables, higher scores consistently reflect better performance or more favorable outcomes (e.g., higher education, better recall, lower task cost, faster reaction times). Solid black lines indicate *p* < 0.05; dashed grey lines indicate *p* ≥ 0.05. For cognitive variable names, see Table 1. For confidence intervals, see: https://osf.io/pu8st/.

**Fig. 5:**
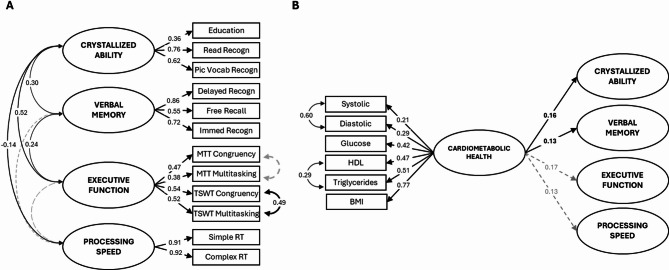
Model 3: Cardiometabolic Health Model. Panel (**A**) shows factor loadings for cognitive factors and their covariances as estimated when examining the association between the Cardiometabolic Health factor and each cognitive factor. Note that there is little difference in loadings compared to Model 1. Panel (**B**) shows standardized factor loadings of the Cardiometabolic Health factor on manifest variables (*Systolic: systolic blood pressure; Diastolic: diastolic blood pressure; HDL: high-density lipoprotein cholesterol; BMI: body mass index*) and standardized path coefficients from the Cardiometabolic Health factor to each cognitive factor. Simple and Complex RT, as well as both Congruency and Multitasking variables were reverse scored to ensure that, for both latent and manifest variables, higher scores consistently reflect better performance or more favorable outcomes (e.g., higher education, better recall, lower task cost, faster reaction times). Solid black lines indicate *p* < 0.05; dashed grey lines indicate *p* ≥ 0.05. For cognitive variable names, see Table 1. For confidence intervals, see: https://osf.io/pu8st/.

### Model 4: Cardiometabolic Health and Cardiorespiratory Fitness (Fig. 6)

**Fig. 6:**
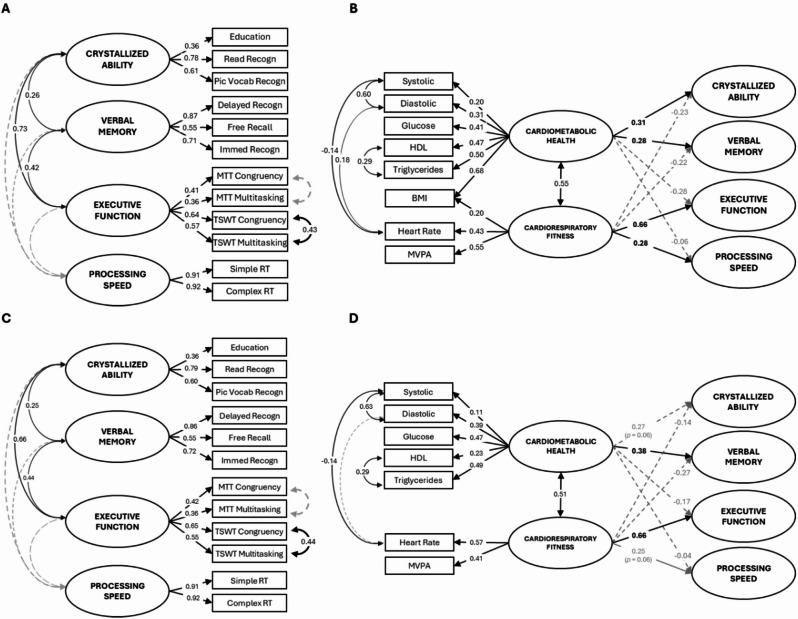
Model 4: Cardiovascular Health Model. This model includes both Cardiorespiratory Fitness and Cardiometabolic Health factors. It shows standardized covariance coefficients of their association and how each factor relates to each cognitive factor, after controlling for their shared variance. Panels (**A**) and **B** present the model with both Cardiorespiratory Fitness and Cardiometabolic Health factors allowed to load on BMI. Panels **C** and **D** present the model without BMI. Simple and Complex RT, as well as both Congruency and Multitasking variables were reverse scored to ensure that, for both latent and manifest variables, higher scores consistently reflect better performance or more favorable outcomes (e.g., higher education, better recall, lower task cost, faster reaction times). Solid black lines indicate *p* < 0.05; dashed grey lines indicate *p* ≥ 0.05. For cognitive variable names, see Table 1. For confidence intervals, see: https://osf.io/pu8st/.

Model 4 includes both cardiovascular health predictors to examine how the above relationships with cognitive outcomes change when controlling for their joint variance (Fig. 6B). As expected, the Cardiorespiratory Fitness and Cardiometabolic Health factors were moderately positively correlated (*β* = 0.55), consistent with a relationship between cardiometabolic health and cardiorespiratory fitness.

When controlling for this relationship between Cardiorespiratory Fitness and Cardiometabolic Health factors, Model 4 not only replicated but strengthened the pattern of prediction of cognitive abilities seen in Models 2 and 3. Specifically, in Model 4, Cardiorespiratory Fitness was more strongly associated with Executive Function (*β* = 0.66) and remained significantly associated with Processing Speed (*β* = 0.28). Once again, it did not significantly predict Crystallized Ability and Verbal Memory.

Likewise, when controlling for shared variance, Cardiometabolic Health was more strongly associated with Crystallized Ability and Verbal Memory factors, with moderate coefficients (*β* = 0.31 and 0.28, respectively), but not with Executive Function or Processing Speed.

### BMI effects

BMI was included in Models 2 and 3, and Model 4 allowed both Cardiometabolic Health and Cardiorespiratory Fitness factors to load onto it. In this joint model (Fig. 6B), it is clear that the Cardiometabolic Health factor loaded much more strongly on BMI than the Cardiorespiratory Fitness factor (*β* = 0.68 vs. 0.20).

To examine whether the correlation between Cardiorespiratory Fitness and Cardiometabolic Health is affected by the inclusion of BMI in both factors and whether this influenced the pattern of relationships with cognitive factors, we reran Model 4 with BMI excluded (Model 4 A, Fig. 6D). Importantly, the strong correlation between Cardiorespiratory Fitness and Cardiometabolic Health remained largely unchanged after removing BMI (*β* = 0.55 to 0.51).

Interestingly, removing BMI from the model did not substantially change the strength of some relationships between cardiovascular factors and cognitive variables, but others now failed to reach conventional level of statistical significance (*p = 0.05*). Specifically, Cardiometabolic Health was still strongly associated with Verbal Memory, but only marginally with Crystallized Ability (*p* = 0.06). Similarly, Cardiorespiratory Fitness was still strongly associated with Executive Function, but only marginally with Processing Speed (*p* = 0.06).

Given that BMI is included in conventional estimations of both cardiometabolic health and cardiorespiratory fitness and that its removal did not substantially change the pattern of findings, we use Model 4 for the remainder of the paper.

### Sex effects

A significant effect of sex was found for both Cardiorespiratory Fitness and Cardiometabolic Health factors [*t*(343) = 2.38, *p* = 0.01 and *t*(343) = -2.07, *p* = 0.03, respectively]. Females had significantly lower Cardiorespiratory Fitness but better Cardiometabolic Health than males.

Sex also moderated the effects of Cardiorespiratory Fitness on Processing Speed [*β* = 0.13, *F*(3,341) = 13.60, *p* < 0.001]. Specifically, while higher Cardiorespiratory Fitness was associated with faster Processing Speed in both sexes, the relationship was only significant in females (*p* < 0.001; males: *p* = 0.08). No other significant moderating effects of sex were found.

## Discussion

In this study, we examined the associations between cardiometabolic health, cardiorespiratory fitness and cognitive abilities across four key domains in a large cohort of cognitively healthy, highly active older adults aged 60–70 yrs. Using SEM, we statistically differentiated the associations of cardiometabolic health and cardiorespiratory fitness with four latent cognitive abilities, after accounting for their shared variance. We selected domains that are differentially impacted by aging: crystallized abilities that improve or remain largely stable at least into late 60s^37^. While previous studies have examined the associations between these cardiovascular health factors and cognitive ability in older adults^34,40,79^, this is the first study to investigate their *unique* and *shared* contributions to specific cognitive domains.

Better cardiometabolic health and cardiorespiratory fitness were both associated with higher cognitive performance in this age-restricted, cognitively healthy older cohort, consistent with existing literature^80–82^. However, they not only showed a distinct pattern of association with different cognitive domains, but evidence consistent with a double dissociation across cognitive domains that remain relatively stable or show little decline with increasing age, and those that are highly sensitive to age-related decline. Specifically, the Cardiorespiratory Fitness factor was significantly associated with Executive Function and Processing Speed factors, but *not* Verbal Memory and Crystallized Ability factors. In contrast, the Cardiometabolic Health factor significantly predicted scores on Crystallized Ability and Verbal Memory factors, but *not* Executive Function and Processing Speed factors. These relationships were observed when each latent cardiovascular factor was modelled independently and persisted, indeed strengthened, in the combined model that accounted for their shared variance.

This is the first study to report a distinct pattern of effects, and indeed evidence consistent with a double dissociation of cardiorespiratory fitness and cardiometabolic health on distinct cognitive domains. Although the observed associations were in the small-to-moderate range (Figs. 3, 4, 5 and 6), they were consistent across model specifications and when using alternative measures (additional models available on OSF, https://osf.io/pu8st/), supporting their robustness and replicability. Moreover, correlations between cardiovascular and cognitive variables of small-to-moderate strength are not unexpected - previous studies have shown that cardiorespiratory fitness and cardiometabolic health are moderately associated with cognitive performance in older adults (e.g., ^74,79,83^). Consistent with this, the sensitivity analysis indicated that the study was adequately powered to detect effects of the magnitude observed, reinforcing confidence in the reliability and stability of the reported associations. We therefore interpret these modest yet consistent effects as reflecting realistic and reproducible relationships between cardiovascular and cognitive systems in later life.

### Cardiorespiratory fitness associated with executive function and processing speed

The strongest association was found between cardiorespiratory fitness and executive function. This relationship was significant across all models and persisted even when the Cardiorespiratory Fitness factor included only heart rate and MVPA. Similar associations have been consistently reported previously^26,84,85^.

Maintaining cardiorespiratory fitness preserves cerebral blood flow and vessel elasticity, slowing or even reversing age-related deterioration in metabolically demanding brain networks and hub regions via metabolic and trophic support^86,87^. These effects are particularly strong in frontal brain regions that are involved in executive functioning and are highly sensitive to vascular and metabolic changes common in older adults^88,89^. For example, higher cardiorespiratory fitness is consistently associated with higher brain volume in frontal and temporal regions^28,90,91^, measures of white matter microstructural organization (e.g., fractional anisotropy, radial diffusivity, myelin water fraction)^92–95^. Additionally, cardiorespiratory fitness modulates blood flow and oxygenation in the prefrontal cortex more than in other brain regions, with oxygenation levels mediating part of the relationship between cardiorespiratory fitness and executive function in high-fit older adults^96–98^.

An association between cardiorespiratory fitness and processing speed has also been reported, albeit less frequently^79,99–101^. The mechanisms are likely similar to those for executive functions. For example, higher cardiorespiratory fitness may slow the progression of age-related vascular changes, thereby protecting white matter microstructural organization^102^, both of which have been shown to predict performance on psychomotor tasks^103–106^.

Despite well-established links between cardiorespiratory fitness, hippocampal structure, and memory performance^107^, in this age-restricted, high functioning older group, we did not find an association between cardiorespiratory fitness and verbal memory. This may be due, at least partly, to the specific memory task used here. The CANTAB Verbal Recognition Memory task assesses immediate word recall and immediate and short-delay recognition memory. These processes rely heavily on short-term memory and less heavily on episodic and working memory, domains that are more susceptible to age-related decline^108^. Bullock et al.^109^ found fitness-related benefits only in high-interference episodic memory tasks that place greater demands on working memory and memory-related inhibition, processes particularly vulnerable to age-related decline^110,111^. A systematic review highlighted the context-dependent nature of fitness-related memory benefits^112^. For instance, Dougherty et al.^29^ showed fitness effects on verbal memory only in males, while Schultz et al.^113^ reported associations with verbal episodic memory exclusively in older adults with high amyloid-beta (Aβ) burden. Longitudinal evidence also suggests that the effects of cardiorespiratory fitness on verbal memory are not as robust as its effects on executive functions and processing speed, after adjusting for age, sex, and education^101,114^. Together, these findings suggest that the relationship between cardiorespiratory fitness and verbal memory in older adults may be weak and more cohort-dependent compared to its effects on other cognitive domains.

### Cardiometabolic health associated with crystallized ability and verbal memory

The Cardiometabolic Health factor loaded consistently on Crystallized Ability and Verbal Memory factors, and these associations were stronger when accounting for its relationship with Cardiorespiratory Fitness (i.e., *β* = 0.13–0.16 to 0.28–0.31). In contrast, it was not significantly associated with executive functions or processing speed.

The relationship between cardiometabolic health and crystallized ability, in the absence of associations with executive functions and processing speed, tentatively suggests that progressive brain vascular deterioration cannot explain this pattern. This is because any cardiometabolic effects on brain vasculature large enough to affect crystallized abilities that are less sensitive to aging, would be expected to have already affected fluid abilities such as executive functioning that are more sensitive to aging^115^.

An alternative interpretation is that this relationship arises from individual variability in socioeconomic status and/or educational attainment in this cohort. The Crystallized Ability factor loaded on years of formal education and two verbal tests from the NIH Toolbox that capture vocabulary size and verbal proficiency – abilities that are widely recognized as proxies for crystallized ability^61,116–118^. Across the lifespan, cardiovascular health is lower for people from lower SES, who often have lower education^119,120^. Low SES is also associated with poorer health literacy, itself linked to worse health outcomes^121,122^. Years of formal education is closely linked to higher SES and predicts improved cardiovascular health and associated mortality^123,124^.

Therefore, a plausible interpretation of the relationship between cardiometabolic health and crystalized ability is that, amongst this age-restricted, community-dwelling cohort, people with lower crystalized ability are more likely to have come from lower SES backgrounds and/or have lower health literacy. These factors are known to contribute to weaker access and/or adherence to healthy lifestyle choices, and may therefore result in poorer cardiometabolic health markers for hypertension, high cholesterol and diabetes^125^.

### Cardiometabolic health not associated with executive function and processing speed

It is surprising that cardiometabolic health was associated with crystalized ability and verbal memory, but it was *not* significantly associated with executive functioning or processing speed. This finding is inconsistent with a strong body of work showing that these cognitive domains are highly susceptible to the presence of cardiovascular disease and risk factors, and particularly hypertension^12,126,127^.

Interestingly, the Cardiometabolic Health factor loaded more on metabolic measures (i.e., lipid profiles, glucose; e.g., *β* = 0.41-0.50 in Model 4) than blood pressure measures (*β* = 0.20-0.31), with systolic blood pressure having the lowest value. The link between blood metabolic markers and executive function ability amongst older adults is weaker than for hemodynamic measures (i.e., systolic blood pressure)^128,129^. Despite blood glucose levels having the strongest loading on the Cardiometabolic Health factor, the cohort’s glucose levels were well within the normal range (Table 2). While Liu et al.^130^ found that blood glucose level was associated with executive functions in non-diabetic older adults, their participants had much higher glucose levels than our cohort.

The largely healthy blood pressure range in our sample may explain why these variables contributed relatively weakly to the Cardiometabolic Health factor, despite the fact that nearly 50% met hypertension criteria and over half were medicated. While the effects of antihypertensive medications on current and future cognitive outcomes are still not clearly established, there is some evidence that pharmacological antihypertensive therapies are associated with reduced dementia risk and selective improvements in attention and executive function^131,132^.

A more plausible explanation for this unexpected finding is that our highly active, cognitively healthy, age-restricted cohort did not have high enough cardiovascular risk to impact higher-order cognition and processing speed, and instead variability in cardiometabolic health was explained by differences in SES or education. As shown in Table 2, our cohort of nearly 350 participants scored, on average, within healthy range for glucose, triglycerides, resting heart rate, HDL, and systolic blood pressure, with obesity levels below cutoff and physical activity well above recommendations. Despite links between cardiometabolic health and crystallized ability, the cohort was highly educated, had SES above the New South Wales average, and cognitive scores well above clinical concern. Recruitment during the COVID-19 pandemic may have led to self-selection of older adults who perceived themselves as less vulnerable, including those with relatively good cardiovascular health.

### Limitations

Importantly, cross-sectional designs limit the ability to draw causal inferences or establish temporal ordering of effects (e.g., lead-lag relationships). As both cardiovascular constructs were derived from physiological and cognitive measures collected contemporaneously, the findings cannot determine how cumulative exposure or changes in cardiovascular health over time influence cognitive functioning. Therefore, while lower cardiorespiratory fitness and poorer cardiometabolic health may contribute to cognitive decline, the direction of these associations cannot be definitively established. Indeed, it is possible that the relationships are bidirectional^133^, whereby emerging cognitive decline leads to reduced physical activity, which in turn lowers fitness and increases cardiometabolic risk in previously healthy individuals. Moreover, some relationships between cardiovascular health and cognition, particularly those involving vascular phenomena such as arteriosclerosis, may be non-linear^134^. Longitudinal or intervention studies are needed to clarify causality and characterize trajectories of change.

It is also important to re-emphasize that this cohort was cognitively high-functioning and physically on the higher end of the health scale. Therefore, our data are more likely to reflect variability related to preservation of function in high-functioning older adults rather than aging-related physical or cognitive decline or dementia risk. While we did not consider other health factors (e.g., diet, sleep quality) that might impact cardiovascular-cognition relationships, the overall healthy metrics for the sample suggest their inclusion may not have impacted the pattern of findings. A strength of this paper was the use of accelerometers, which allowed for the objective measurement of physical activity. It is, however, acknowledged that physical activity outcomes can differ depending on the device placement (e.g., wrist or hip), choice of epoch length used for analysis (e.g., 60 s), and the choice of metrics (e.g., total time or fragmentation indices) cut points used. Here we used cut-points for MVPA described in Hilderbrand et al.^135^ which although were developed in a study population younger than ours, have been used in similar studies by our group and others with active older adult samples^136–138^.

Finally, the study also involved a moderate level of participant exclusion (19% of participants excluded; see Fig. 2). This could potentially introduce bias, as participants retained for analysis may differ systematically from those excluded (e.g., in health status, cognitive ability, testing compliance). However, such exclusion rates are typical in large, community-based studies involving extensive testing across multiple sites. Importantly, the pattern of results was highly consistent between the complete-data subsample (*n* = 223) and the larger analytic sample that included participants with incomplete data (*n* = 345, modeled using FIML), supporting the robustness of these findings.

### Conclusion and future directions

This is the first study to show evidence consistent with a double dissociation between the effects of cardiorespiratory fitness and cardiometabolic health on different cognitive domains in a large, cross-sectional, age-restricted, high-functioning older cohort. Specifically, cardiorespiratory fitness was strongly associated with executive function and processing speed but not crystallized ability or verbal memory; conversely, cardiometabolic health was strongly linked to crystallized ability and verbal memory but not executive function or processing speed. We interpret the lack of association between cardiometabolic health and fluid ability tasks as partly due to our cohort’s generally healthy cognitive and cardiometabolic status. However, it is important to note that, even in this healthy cohort, cardiorespiratory fitness remained strongly linked to executive function and processing speed. Future research should test whether this pattern of double dissociation generalizes to younger or more heterogeneous populations, including individuals with a broader range of socioeconomic and health backgrounds. Such work would help clarify whether the relationships observed here reflect age-specific mechanisms that emerge in later life or more general physiological–cognitive couplings that operate across the adult lifespan. Longitudinal or lifespan-comparative studies integrating both younger and older cohorts, and encompassing greater variability in socioeconomic status and health profiles, would be particularly valuable for determining whether cardiorespiratory fitness and cardiometabolic health differentially support distinct cognitive domains at different stages of adulthood.

Overall, these findings support a healthy aging framework that emphasizes prevention and lifestyle-based health promotion. Importantly, they suggest that different strategies may confer distinct cognitive benefits. Improving cardiorespiratory fitness (for instance, through structured aerobic exercise and active living initiatives) appears particularly beneficial for maintaining executive function and processing speed. Conversely, we argue that the association between cardiometabolic health and crystallized ability and verbal memory is more likely to reflect an intervening effect of health literacy regarding dietary interventions, glucose and lipid regulation, and cardiovascular risk monitoring. These results highlight the need for multifaceted public health programs that address both dimensions of cardiovascular health. Such initiatives should go beyond clinical treatment models to include accessible community programs that combine physical activity, nutrition education, and health literacy interventions aimed at sustaining both physical and cognitive vitality across aging.

## Data Availability

Due to the ongoing longitudinal nature of the ACTIVate study, the dataset analyzed during this study is not publicly available. However, it may be obtained from the corresponding author upon reasonable request. The main analysis code and supplementary results are available at the OSF repository: [https://osf.io/pu8st/].
